# Gaps in Hepatitis B Vaccination Completion and Sero-Protection for People Who Inject Drugs in Hpakant, Myanmar, 2015–2018

**DOI:** 10.3390/tropicalmed5020077

**Published:** 2020-05-12

**Authors:** Nilar Shwe Yee, Aung Yu Naing, Julita Gil Cuesta, Mrinalini Das, Kapilkumar Dave

**Affiliations:** 1 Asian Harm Reduction Network, Hpakant Township, Kachin State 01161, Myanmar; aungyunaing@ahrnmyanmar.org; 2 Luxembourg Operational Research Unit, Médecins Sans Frontières, 1050 Brussels, Belgium; Julita.GIL@luxembourg.msf.org; 3 Médecins Sans Frontières (MSF)/Doctors Without Borders, New Delhi 110001, India; MSFOCB-Delhi-Epi@brussels.msf.org; 4 SEWA Rural, Jhagadia, Bharuch district, Gujarat 393110, India; Kapil.dave88@gmail.com

**Keywords:** hepatitis B vaccination, sero-protection, people who inject drugs, SORT-IT

## Abstract

Hepatitis B vaccination (HBV) is recommended for high-risk groups, such as people who inject drugs (PWIDs). As part of a harm reduction program by a non-governmental organization, hepatitis B screening, vaccination and antibody (HBAb) testing after completion of the vaccination schedule were offered to PWIDS in Myanmar. We determined the proportions of HBV non-completion and sero-unprotection among PWIDs enrolled in the program and their association with socio-demographic and clinical characteristics. We conducted a descriptive study based on routine program data in five selected clinics in Hpakant Township, Myanmar. PWIDs who were Hepatitis B antigen negative at screening during January 2015–December 2018 were included. Among 5386 participants eligible for HBV, 9% refused vaccination. Among those who accepted vaccination (*n* = 3177 individuals), 65% completed vaccination. Of those tested for HBsAb (*n* = 2202), 30% were sero-unprotected. Young-adults (aged 18–44 years) and migrant workers had a higher risk of incomplete vaccination. However, participants who used methadone had a lower risk of incomplete vaccination. Migrant workers had higher risk of not returning for HBsAb testing and HIV-positive participants had a higher risk of being HBV sero-unprotected. Efforts to increase HBV vaccination in PWIDs for young adults and clients during methadone and anti-retroviral services should be prioritized.

## 1. Introduction

In South-East Asia there were an estimated 39 million people living with chronic hepatitis B viral infection in 2015 [1]. Among adults, ongoing HBV transmission occurs primarily among incompletely immunized clients with behavioural risks for HBV transmission including individuals with multiple sex partners, people who inject drugs (PWIDs), men who have sex with men and household contacts and sex partners of persons with chronic HBV infection. Hepatitis B vaccination is an effective measure to prevent HBV infection and its consequences, including cirrhosis of the liver, liver cancer, liver failure and death [2].

In Myanmar, the prevalence of viral hepatitis in the general population was 10%−12% in 2013, and viral hepatitis places a heavy burden on the health care system because of the costs of treatment of liver failure and chronic liver disease [3]. The prevalence of HIV/HBV co-infection was found to be 3.2% and HIV/HCV co-infection was 26.8%, in Myanmar in 2019 [4]. Myanmar has experienced an increase in illicit drug use problems in the last decade, especially in the population of 15−49-year-olds. There were an estimated 93,000 people who inject drugs (PWIDs) in 2017 who accessed the prevention and medical intervention services in Myanmar [4].

Previous studies in Myanmar have shown a 7%−8.7% HBV burden [5]. The sero prevalence of hepatitis B being 8.7%, and the co-infection with HIV 0.35%. HIV men who have sex with men are at the highest risk of being co-infected with hepatitis B while intravenous drug users are at the highest risk of being co-infected with hepatitis C [5].

The Asian Harm Reduction Network (AHRN), a non-government organization, provides harm reduction support services to vulnerable populations such as PWIDs (*n* = 32,800) in the Kachin, Sagaing and Northern Shan states of Myanmar. Hpakant township is one such place with PWIDs (*n* = 14,462) in 2018.

The AHRN services provided a unique opportunity to access PWID—one of the known high risk groups for HBV—and to evaluate the HBV preventive services offered to PWIDs in the program. We therefore conducted a study to assess the Hepatitis B vaccination completion and sero-conversion rates among PWID according to socio-demographic characteristics and HIV status in the Hpakant township, Kachin State, Myanmar.

The results of the study identified the gaps in hepatitis B vaccination completion and sero-protection among PWIDs in the Hpakant Township, Myanmar.

## 2. Materials and Methods

### 2.1. Study Design

This was a descriptive study based on routine program data from 2015 to 2018.

### 2.2. Setting

The study was conducted in the Hpakant township, Kachin state, Myanmar. It is one of the jade mining areas, with a total general population of 312,300 [6]. It is located in Mohnyin district, and divided into 15 village tracts and 116 villages [6]. An estimated 115,300 PWIDs are accessing the prevention services [4].

#### 2.2.1. Myanmar National Hepatitis Control Program

The National Hepatitis Control Program has developed a specific national strategy and policy for community and hospital infection control. They include equity, universal health coverage, a public health approach, evidence-based policy and program planning, partnership and community engagement and health care precautions to prevent hepatitis B and hepatitis C infections in health-care settings [7].

#### 2.2.2. Asian Harm Reduction Network (AHRN) Hepatitis B Vaccination Program for PWIDs

Asian Harm Reduction Network (AHRN) Myanmar is an international non-governmental organization implementing a harm reduction program to reduce the transmission of HIV/AIDS, TB and other blood-borne diseases among PWID/PWUD, their families/partners and their community members from 2003. Services provided in Hpakan consist of prevention and medical intervention services through outreach and mobile clinics, Needle and Syringe Programs, condom distribution, educational materials distribution, HIV testing service, antiretroviral treatment, screening and treatment of sexually transmitted infections, screening and vaccination for viral hepatitis B, screening and treatment for viral hepatitis C, screening and treatment of tuberculosis, overdose prevention and management, post-exposure prophylaxis, psychosocial support, drug adherence counseling and transportation allowance.

In the AHRN Program, PWIDs receive health education about hepatitis and are offered a referral for HBV treatment. If clients are HBV negative during screening (HBV antigen test), they are started on an accelerated vaccination schedule at days 0, 7 and 21 [8]. The vaccine dosage for HBV is 20 μg for HIV negative and 40 μg for HIV positive clients. Antibodies for hepatitis B are tested two months after the third vaccination. AHRN provides free medical care support to all clients enrolled in the program. An incentive of 2000 Kyats (1.4 USD) is given to PWIDs after completion of the third dose vaccination.

The clinic nurse offers PWID clients HIV counseling and testing, and hepatitis B screening. If they are hepatitis B negative, the clinic nurse asks them whether they are willing to receive the vaccination, which is then administered.

### 2.3. Study Site and Population

Five clinics (Hpakant, Seik Mu, Lone Khin, Tamakhan and Selzin) in the Hpakant Township were selected for the study. These sites were chosen based on a high number of PWIDs and study feasibility. We included all PWID clients who were eligible to receive hepatitis B vaccination (HBV antigen-negative), enrolled in the program between 1 January 2015 and 31 December 2018 in the five study sites. Clients who were hepatitis B antigen positive during screening, and those who had been previously vaccinated were excluded.

### 2.4. Operational Definitions

- PWID: People who injected any illicit drugs at least once in the past 12 months. Injections might be intramuscular, subcutaneous and intravenous.
- Eligibility for HBV vaccination: PWIDs who tested negative for HBs Antigen (surface antigen of the hepatitis B virus) screening in the AHRN clinic.
- HBV vaccination completion: PWIDs who had received the three doses of vaccine. Vaccination status was verified through the vaccination card.
- HBV sero-protection: Anti-HBs tests were performed two months after the third dose of hepatitis B vaccine. If adequate anti-HBs were present (>10 mIU/mL), clients were considered sero-protected. Periodic testing or boosting was not performed.
- HBV sero-non-protection: PWIDs were defined as HBV sero-unprotected if they had completed the third dose but were HBV antibody negative on retesting two months after the third dose.
- Educational level:
  ◦ Illiterate: a person who was unable to read and write;
  ◦ Primary: a person who attended the first stage of basic education, and was able to read and write;
  ◦ Middle: a person who had passed standard 8;
  ◦ High: a person who had been enrolled to standard 9 or 10;
  ◦ University: a person who had been enrolled in any Myanmar university.
- HIV unknown status: a person who refused to test for HIV at the time of hepatitis B screening [8].
- HBs Antigen Test: The SD Bioline HBsAg WB test was used for qualitative determination of HBsAg status [9].
- Hepatitis B vaccine (rDNA) was a non-infectious recombinant DNA hepatitis B vaccine [10].
- The DIAQUICK HBs Ab Dipstick, a rapid chromatographic lateral flow immunoassay for the qualitative detection of antibody to HBs antigen in serum or plasma, was used as the marker of immunity to HBV [11].

### 2.5. Data Variables, Sources of Date and Data Collection

The variables included the project site, client identification number, type of client, date and result of HBsAg, HBV vaccination accepted or not, HIV status, gender, age, risk group (PWIDs only, PWID who used methadone, PWID who were commercial sex workers), date of first, second, third doses, dosage and HBV antibody results that were recorded in the AHRN program electronic database. They were extracted and reviewed with the program officer to ensure completeness and quality of the data. A vaccination card is started and clients are given an appointment for the next vaccination and checked the completion of vaccination card and gave the next date appointment. The clinic nurse entered this information into a paper-based format. Then, the data assistant entered it into the access based software and checked the data for monthly reporting in the study clinic.

### 2.6. Data Analysis and Statistics

Data was exported into EpiData analysis software (version 2.2.2.183) and STATA (version 11, StataCorp, College Station, TX, USA) for data analysis. Continuous variables such as age were described as median and interquartile ranges (IQR). Categorical variables such as gender (male/female), vaccination completion (yes/no) and sero-protection (yes/no) were described as frequencies and proportions. Unadjusted risk ratios (RR) and adjusted risk ratios (aRR) were calculated for the association of select socio-demographic and clinical factors (age group, gender, project site, educational levels, occupation, risk group and HIV status) with outcome variables (HBV vaccination refusal, vaccination incompletion, those who did not return for HBV antibody testing and HBV sero-un-protection). Confidence intervals (CI) of 95% and a *p*-value < 0.05 were considered significant.

### 2.7. Ethics

Ethics approval was obtained from the Institutional Review Board, Department of Medical Research, Ministry of Health and Sports, Myanmar (Ethics/DMR/2019/132), the Ethics Advisory Group (EAG) of the Union, Paris (25/19), the international relation division (2019/1248) from the Ministry of Health and Sports, Myanmar, and National Drug Dependency Treatment and Research Unit, Myanmar.

## 3. Results

**Figure 1:**
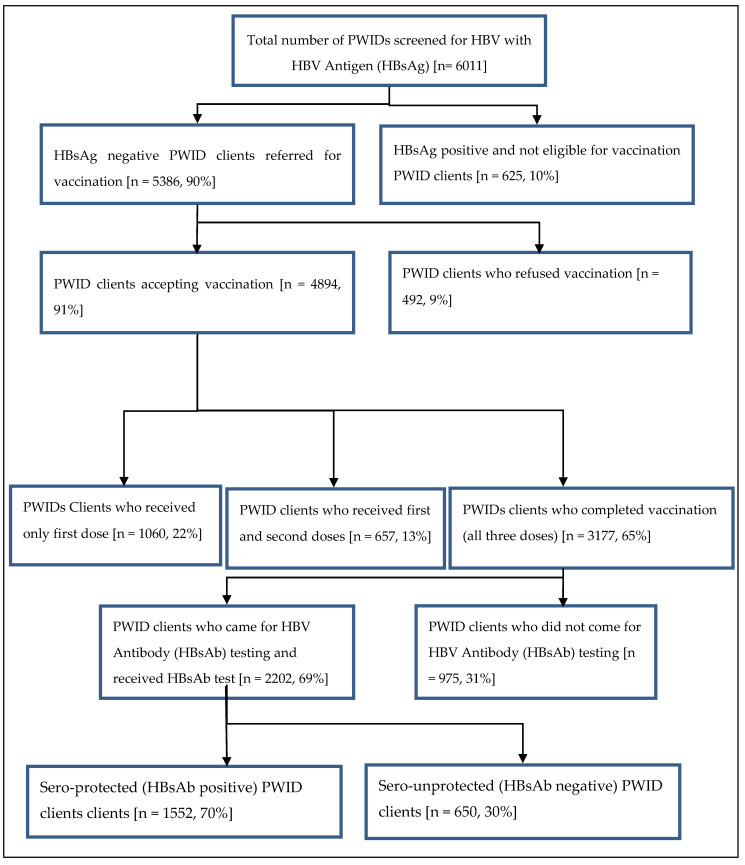
Flow of HBV vaccination completion among PWID clients in Hpakant Township, Kachin State, Myanmar between January 2015 and December 2018.

A total of 6011 PWIDs were screened for HBV antigen (HBsAg) during the study period (Figure 1). Of those screened, 10% were HBV infected (625 HBsAg positive); thus, vaccination was not required. Among the 5386 PWIDs who were eligible for HBV vaccination, 89% were 18–44 years of age, 10% 45–64 years of age and 1% 65–75 years of age. Nine percent (492/5386) refused the vaccination. Among those who accepted the vaccination, 22% received only one vaccine dose, 13% received two doses and 65% three doses (complete vaccination). Among those completely vaccinated, 31% (975) of the PWIDs did not return for HBs antibody testing after the third dose. Of those who returned and were tested with the HBV antibody (HBsAb), 30% (650) were sero-unprotected. Clients who belonged to Hpakant—aRR (95%CI): 1.6 (1.2–2.2), Seng Taung—aRR (95%CI): 1.8 (1.4–2.3) and Lone Khin—aRR (95%CI): 1.8 (1.4–2.3) had a higher risk of not returning for HBs Ab testing, while those from Tamakhan—aRR (95%CI): 0.6 (0.5−0.9) had a lower risk compared to those who lived in Selzin. Among all recorded occupations, migrant workers—aRR (95%CI): 1.3 (1.0–1.6) had a higher risk of not coming for HBs Ab testing.

### 3.1. Characteristics of Participants According to HBV Vaccination Refusal

The socio-demographic and clinical characteristics of PWIDs eligible for HBV vaccination (i.e., those who were HBsAg negative on screening) are described in Table 1. The majority (89%) were aged between 18 and 44 years and were males (98%). About 34% were residents of Seng Taung followed by Lone Khin (29%). 55% had completed high school, and the most frequent occupation was being a farmer (45%). About 27% of PWIDs also used methadone and 39% had HIV infection. PWIDs with residence in Hpakant (aRR: 2.3; 95% CI: 1.3–3.9) or Lone Khin (aRR: 2.3; 95%CI: 1.3–3.8) were more likely to refuse vaccination as compared with Selzin. Participants with HIV results had a lower likelihood for refusal to vaccination than the ones with unknown HIV status (HIV positive: (aRR (95%CI): 0.4 (0.4–0.6) and negative: (aRR (95%CI): 0.2 (0.1–0.3)). Among those who accepted vaccination, 22% received only one vaccine dose, 13% received two doses and 65% three doses (complete vaccination).

### 3.2. Factors Associated with HBV Sero-Unprotection

Table 2 shows the socio-demographic and clinical characteristics associated with PWIDs who did not come for HBs Ab testing after completion of HBV vaccination. Clients who belonged to Hpakant (aRR (95%CI): 1.6 (1.2–2.2)), Seng Taung (aRR (95%CI): 1.8 (1.4−2.3)), Lone Khin (aRR (95%CI): 1.8 (1.4–2.3)) had a higher risk of not returning for HBs Ab while PWIDs from Tamakhan (aRR (95%CI): 0.6 (0.5–0.9)) had a lower risk compared to those who lived in Selzin. Among all recorded occupations, migrant workers (aRR (95%CI): 1.3 (1.0–1.6)) had a higher risk of not coming for HBs Ab testing.

Among participants with completed vaccination in which HBsAb test was done, factors associated with sero un-protection are shown in Table 3. HIV positive participants had a higher risk of being HBV sero-unprotected compared to the ones with HIV negative status [aRR (95%CI): 1.8 (1.6–2.2)].

## 4. Discussion

In our study, one third of PWIDs did not complete the hepatitis B vaccination schedule. Among those who completed the vaccination and had their Hepatitis B Antibody performed, almost one third were sero-unprotected. However, the fact that two-thirds of PWIDs clients completed hepatitis B vaccination demonstrated the performance of our harm reduction program for PWIDs. It compares favorably with hepatitis B vaccination programs elsewhere: 52% completion rates in UK [12], 47%, 31% and 27% in different US settings [13] and 59.2% of PWIDs who had received a first dose in Sweden [14].

We found several socio-demographic and clinical characteristics associated with incomplete vaccination and sero-un-protection. We present them separately to tackle each of the findings relevance for programmatic reorientations.

In the AHRN program, HIV and HBV screening were offered at the same place on the same day, which may have helped PWIDs receive information about both HIV and HBV. However, one-tenth of PWIDs refused HIV testing, which may underestimate the proportion of HIV in the study population. Our study reported clients with HIV infection to be more likely HBV sero-unprotected. This indicates that despite the higher vaccination dose used in HIV positive PWIDs, we need to continue our efforts to offer and comply with the second series of three doses, as mentioned in the literature [15].

More than one-third of young and middle-aged adults (aged 18–44 years) failed to complete the HBV vaccination. This finding is in line with a recent study in London that mentioned that PWIDs in a similar age group were less likely to complete vaccination [12]. Considering that this age group is economically active and may incur a loss of wages, they may have more trouble coming for multiple visits to complete vaccination. Though the program provides monetary incentives after completion of the third dose, additional transport reimbursements on the day of the first and second doses might motivate clients to return for vaccination. It has been recognized that adults with high-risk behaviours, such as PWIDs, are the second most urgent group to be prioritized for complete vaccination after infants [16], thus this group needs attention.

Migrant workers are known for being lost to follow up in vaccination programs [17]. Our study found that a significant proportion of migrant workers did not return for HBV antibody testing after completion of vaccination. To address this, programs must devise strategies to establish a referral link to other program sites or government healthcare centers for migrant workers, near their places of work. Catch-up vaccination drives using mobile vans that visit jade mining sites would help to complete vaccination schedules.

In our study, PWIDs who were using methadone had a higher proportion of vaccination completion. More than half of PWIDs using methadone were HIV-infected, and they received ART services in the same clinic. We suggest that, as methadone users were coming to the clinic often to receive methadone and ART services, they were more likely to complete the vaccination schedule [18]. Methadone users could be great advocates for HBV vaccination and inform their networks to avail the services of the clinic.

The study had the following strengths. HBV vaccination services were evaluated for PWIDs in the routine program of AHRN in Myanmar and thus reflect the on-the-ground realities. Our study population was high risk for HBV and difficult to reach, and therefore the study created a unique opportunity to access those clients through AHRN services. The program activities of HBsAg screening, consent for vaccination, vaccination provision, HBsAb testing and data entry were performed by trained staff. Vaccination cards were checked by the nurse on the day of antibody testing to confirm vaccination completion [19].

Our study had three main limitations. It was based on secondary data, so there could be errors and omissions, though the data supervisor in the program ensured the quality of data. The study included only clients of the AHRN services, thus the results of the study may not be generalizable to all PWIDs in Myanmar. PWIDs attending AHRN may have different attitudes and adherence behaviours towards health services than other PWIDs not attending. Approximately one-tenth of PWIDs refused to do an HIV test, so the proportions of HBV sero-non-protection in HIV positive PWIDs may be under-estimated.

## 5. Conclusions 

In a harm-reduction program for PWIDs in Myanmar, we demonstrated that two-thirds of clients completed the three injection vaccination schedule, and of those, 70% showed sero-conversion. However, one-third of the most numerous and economically active group of 18−44-year-old age group failed to complete the vaccination schedule. As well, migrant workers had a poorer completion rate and HIV clients had a lower sero-conversion rate despite a higher dose of vaccine. We recommend HBV programs in this setting should focus on young adults and migrant workers as priorities for completion of vaccination. Methadone users receiving HIV services may also be a good resource in recruiting clients for hepatitis B vaccination.

## Figures and Tables

**Table 1:** Socio-demographic and clinical characteristics associations with PWIDs eligibility for HBV vaccination (HBsAg negative) and vaccination refusal in the Hpakant Township, Kachin State, Myanmar, January 2015 and December 2018.

Socio-Demographic Characteristics		PWIDs Eligible for HBV Vaccination (HBsAg Negative) *		PWIDs Who Refused HBV Vaccination **			
		*N*	(%)	*N*	(%)	aRR (95%CI)	*p*-Value
**Total**		**5386**	**(100)**	**492**	**(9)**		
**Age group**							
	**18−44**	4773	(89)	431	(9)	0.8 (0.6–1.0)	0.11
	**45−64**	602	(10)	61	(10)	Ref	
	**65−75**	11	(1)	−	−	−	−
**Gender**							
	**Male**	5295	(98)	487	(9)	1.5 (0.5–3.9)	0.41
	**Female**	91	(2)	5	(5)	Ref	
**Project site**							
	**Hpakant**	905	(17)	116	(13)	2.3 (1.3–3.9)	<0.01
	**Seng Taung**	1832	(34)	152	(8)	1.8 (1.1–3.0)	0.01
	**Lone Khin**	1556	(29)	166	(11)	2.3 (1.3–3.8)	<0.01
	**Tamakhan**	683	(13)	39	(6)	1.2 (0.6–2.1)	0.54
	**Selzin**	410	(8)	19	(5)	Ref	
**Educational level**							
	**Illiterate**	324	(6)	27	(8)	1.1 (0.7–1.7)	0.56
	**Primary**	1316	(24)	113	(9)	1.2 (0.7–2.0)	0.48
	**Middle**	322	(6)	25	(8)	1.0 (0.6–1.8	0.91
	**High**	2976	(55)	301	(10)	1.3 (0.8–1.9)	0.17
	**University level**	448	(8)	26	(6)	Ref	
**Occupation**							
	**Drug dealer ^1^**	225	(4)	15	(7)	0.9 (0.5–1.7)	0.99
	**Driver**	220	(4)	24	(11)	1.2 (0.7–1.9)	0.45
	**Farmer**	2406	(45)	198	(8)	0.8 (0.6–1.2)	0.41
	**Jade broker ^2^**	575	(11)	54	(9)	1.3 (0.8–1.9)	0.19
	**Migrant worker**	636	(12)	58	(9)	0.9 (0.6–1.3)	0.65
	**Daily worker**	886	(16)	103	(12)	1.0 (0.7–1.4)	0.76
	**Mining worker**	438	(8)	40	(9)	Ref	
**Clinical characteristics**							
**Additional risks**	**PWID**	3943	(72)	414	(10)	0.8 (0.1–6.9)	0.85
	**PWID who also used Methadone**	1432	(27)	77	(5)	0.3 (0.0–3.1)	0.36
	**PWID who were Commercial Sex workers**	11	(1)	1	(9)	Ref	
**HIV**							
	**Positive**	2098	(39)	219	(10)	0.4 (0.4–0.6)	<0.01
	**Negative**	2746	(51)	139	(5)	0.2 (0.1–0.3)	<0.01
	**Unknown**	542	(10)	134	(25)	Ref	

* Column percentage, ** row percentage, refused vaccination: a person who did not want to take three doses vaccination due of the high cost of transportation, ^1^ drug dealer: a person who sells illicit drugs, ^2^ jade broker: a person who buys and sells a green precious stone and jewelry, migrant worker: a person who lives away from their town or village of origin, mining worker: a person who works in the mining areas, *N* = number of case, aRR = adjusted risk ratio, CI = confidence interval.

**Table 2:** Socio-demographic and clinical characteristics associations with PWIDs HBV antibody testing after completion of HBV vaccination in the Hpakant township, Kachin state, Myanmar between January 2015 and December 2018.

Characteristics		Total	PWIDs Clients Who Did Not Come for HBs Ab Testing *			
Socio-Demographic Characteristics		*N*	*N*	(%)	aRR (95%CI)	*p*-Value
	**Total**	**3177**	**975**	**(31)**		
**Age group**						
	**18–44**	2744	872	(32)	1.1 (0.9–1.3)	0.06
	**45–64**	422	101	(24)	Ref	
	**≥65**	11	2	(18)	0.8 (0.3–2.7)	0.82
**Gender**						
	**Male**	3111	963	(31)	1.6 (0.9–2.9)	0.06
	**Female**	66	12	(18)	Ref	
**Project site**						
	**Hpakant**	532	157	(30)	1.6 (1.2–2.2)	<0.01
	**Seng Taung**	1040	363	(35)	1.8 (1.4–2.3)	<0.01
	**Lone Khin**	905	315	(35)	1.8 (1.4–2.3)	<0.01
	**Tamakhan**	421	70	(17)	0.6 (0.5–0.9)	0.02
	**Selzin**	279	70	(25)	Ref	
**Educational level**						
	**Illiterate**	183	56	(31)	0.9 (0.7–1.1)	0.43
	**Primary**	767	222	(29)	1.0 (0.8–1.4)	0.58
	**Middle**	209	55	(26)	0.8 (0.6–1.1)	0.33
	**High**	1714	561	(33)	1.0 (0.8–1.2)	0.92
	**University level**	304	81	(27)	Ref	
**Occupation**						
	**Farmer**	1370	412	(30)	1.0 (0.8–1.2)	0.71
	**Daily worker**	560	159	(28)	1.0 (0.8–1.3)	0.74
	**Migrant worker**	364	137	(38)	1.3 (1.0–1.6)	0.02
	**Jade broker ** ^**2**^	352	126	(36)	1.2 (0.9–1.5)	0.14
	**Mining worker**	260	72	(28)	Ref	
	**Drug dealer ** ^1^	146	32	(22)	1.1 (0.7–1.6)	0.53
	**Driver**	125	37	(30)	1.2 (0.9–1.7)	0.17
**Clinical characteristics**						
**Type of client**						
	**PWID**	2028	733	(36)	0.6(0.1–2.4)	0.57
	**PWID who also used Methadone**	1144	240	(21)	0.3(0.0–1.1)	0.07
	**PWID who were Commercial Sex workers**	5	2	(40)	Ref	
**HIV**						
	**Positive**	1205	388	(32)	0.9(0.7–1.1)	0.52
	**Negative**	1701	487	(29)	0.8(0.7–1.0)	0.08
	**Unknown**	271	100	(37)	Ref	

* Row percentage, ^1^ drug dealer: a person who sells illicit drugs, ^2^ jade broker: Jade broker: a person who buys and sells a green precious stone and jewellery, migrant worker: a person who lives away from their town or village of origin, mining worker: a person who works in the mining areas, *N* = number of case, aRR = adjusted risk ratio, CI = confidence interval.

**Table 3:** Sero-unprotection status according to socio-demographic and clinical characteristics in the Hpakant township, Kachin state, Myanmar between January 2015 and December 2018.

Characteristics		Total	Sero-Unprotected Clients HBsAntibody Negative) *			
Socio-Demographic Characteristics		*N*	*N*	(%)	aRR (95%CI)	*p*-Value
	**Total**	**2202**	**650**	**(30)**		
**Age group**						
	**18–44**	1872	549	(29)	0.8 (0.3–2.0)	0.76
	**45–64**	321	98	(31)	0.9 (0.3–2.1)	0.81
	**≥65**	9	3	(33)	Ref	
**Gender**						
	**Male**	2148	633	(29)	0.9 (0.6–1.4)	0.69
	**Female**	54	17	(31)	Ref	
**Project site**						
	**Hpakant**	375	110	(29)		
	**Seng Taung**	677	145	(21)		
	**Lone Khin**	590	222	(38)		
	**Tamakhan**	351	94	(27)		
	**Selzin**	209	79	(38)		
**Educational level**						
	**Illiterate**	127	31	(24)	0.9 (0.7–1.2)	0.95
	**Primary**	545	169	(31)	0.7 (0.5–1.1)	0.18
	**Middle**	154	57	(37)	1.1 (0.8–1.5)	0.39
	**High**	1153	325	(28)	0.9 (0.7–1.1)	0.37
	**University level**	223	68	(30)	Ref	
**Occupation**						
	**Farmer**	959	274	(29)	0.8 (0.6–1.0)	0.09
	**Daily worker**	401	109	(27)	0.8 (0.6–1.0)	0.12
	**Migrant worker**	227	76	(33)	0.9 (0.7–1.2)	0.88
	**Jade broker ** ^2^	226	65	(29)	0.8 (0.6–1.1)	0.24
	**Mining worker**	188	65	(35)	Ref	
	**Drug dealer ** ^1^	114	34	(30)	0.9 (0.6–1.2)	0.58
	**Driver**	87	27	(31)	1.0 (0.7–1.4)	0.93
**Clinical characteristics**						
**Type of client**	**PWID**	1295	371	(29)	0.6 (0.2–2.3)	0.51
	**PWID who also used Methadone**	904	278	(31)	0.6 (0.2–2.3)	0.54
	**PWID who were Commercial Sex workers**	3	1	(33)	Ref	
**HIV**						
	**Positive**	817	344	(42)	1.9 (1.6–2.2)	<0.01
	**Negative**	1214	270	(22)	Ref	
	**Unknown**	171	36	(21)	0.9 (0.7–1.3)	0.85

* Row percentage, refused vaccination: a person who did not want to take three doses vaccination due of the high cost of transportation, 1 drug dealer: a person who sells illicit drugs, ^2^ jade broker: a person who buys and sells a green precious stone and jewellery, migrant worker: a person who lives away from their town or village of origin, mining worker: a person who works in the mining areas, *N* = number of case, aRR = adjusted risk ratio, CI = confidence interval.
